# The Non-peptide Angiotensin-(1–7) Mimic AVE 0991 Attenuates Delayed Neurocognitive Recovery After Laparotomy by Reducing Neuroinflammation and Restoring Blood-Brain Barrier Integrity in Aged Rats

**DOI:** 10.3389/fnagi.2021.624387

**Published:** 2021-02-15

**Authors:** Xinning Mi, Yiyun Cao, Yue Li, Yitong Li, Jingshu Hong, Jindan He, Yaoxian Liang, Ning Yang, Taotao Liu, Dengyang Han, Chongshen Kuang, Yongzheng Han, Yang Zhou, Yajie Liu, Chengmei Shi, Xiangyang Guo, Zhengqian Li

**Affiliations:** ^1^Department of Anesthesiology, Peking University Third Hospital, Beijing, China; ^2^Department of Anesthesiology, The Sixth People’s Hospital, Shanghai Jiao Tong University, Shanghai, China; ^3^Department of Nephrology, Peking University People’s Hospital, Beijing, China

**Keywords:** delayed neurocognitive recovery, AVE 0991, renin-angiotensin system, neuroinflammation, blood-brain barrier

## Abstract

Delayed neurocognitive recovery (dNCR) after surgery is a common postoperative complication in older adult patients. Our previous studies have demonstrated that cognitive impairment after surgery involves an increase in the brain renin-angiotensin system (RAS) activity, including overactivation of the angiotensin 2/angiotensin receptor-1 (Ang II/AT1) axis, which provokes the disruption of the hippocampal blood-brain barrier (BBB). Nevertheless, the potential role of the counter-regulatory RAS axis, the Ang-(1–7)/Mas pathway, in dNCR remains unknown. Using an aged rat model of dNCR, we dynamically investigated the activity of both axes of the RAS following laparotomy. AVE 0991, a nonpeptide analog of Ang-(1–7), was administered intranasally immediately after laparotomy. We found that the elevation of Ang II, induced by surgery was accompanied by a decrease of Ang-(1–7) in the hippocampus, but not in the circulation. Surgery also significantly downregulated hippocampal Mas receptor expression at 24 h postsurgery. Mas activation with intranasal AVE 0991 treatment significantly improved hippocampus-dependent learning and memory deficits induced by surgery. Furthermore, it attenuated hippocampal neuroinflammation, as shown by the decreased level of the microglial activation marker cluster of differentiation 11b (CD11b) and the decreased production of several inflammatory molecules. Along with these beneficial effects, the AVE 0991 treatment also alleviated the imbalance between matrix metalloproteinase-9 (MMP-9) and tissue inhibitor of matrix metalloproteinase-3 (TIMP-3), modulated the expression of occludin, and alleviated the IgG extravasation, thereby restoring the integrity of the BBB. In conclusion, these data indicate that activation of Mas by AVE 0991 attenuates dNCR after surgery by reducing neuroinflammation and restoring BBB integrity. Our findings suggest that the Ang-(1–7)/Mas pathway may be a novel therapeutic target for treating dNCR after surgery in older adult patients.

## Introduction

With the development of anesthetic and surgical technologies and the rapid growth of the aging population, an increasing number of older adult patients are receiving surgical treatments under anesthesia. Unfortunately, the peripheral surgical trauma inevitably triggers neuroinflammatory processes, meaning that older adult patients are more prone to suffering delayed neurocognitive recovery (dNCR) after surgery because of central nervous system (CNS) degeneration (Evered et al., 2017). This condition mainly manifests as cognitive impairment, memory loss, mental disorder, and social impairment, and may even lead to Alzheimer’s disease (AD) in some severe cases, causing a heavy medical and social burden (Deiner and Silverstein, 2009). The pathological mechanisms underlying dNCR are very complicated, with recent studies showing that the occurrence of postoperative dNCR is associated with a range of deleterious effects, including neuroinflammation, blood-brain barrier (BBB) disruption, oxidative stress, and central cholinergic deficiencies (Terrando et al., 2011; He et al., 2012). Given the lack of understanding of the underlying pathological mechanisms, preventing or treating dNCR can be difficult.

The renin-angiotensin system (RAS) is composed of circulating and local components (Ganten et al., 1971), and plays important roles in regulating blood pressure and volume. In addition to its role in the central regulation of blood pressure and water-electrolyte balance, the local RAS in the brain is also involved in a variety of neurological diseases. Angiotensin 2 (Ang II) *via* its angiotensin receptor-1 (AT1) is found to contribute deleteriously to neuroinflammation and oxidative stress, reduced cerebral blood flow, tissue remodeling, disruption of memory consolidation and retrieval (Wright and Harding, 2019). As a degradation metabolite of Ang II, angiotensin-(1–7) [Ang-(1–7)], has been shown to counter the damaging influences of Ang II, exerts a neuroprotective effect in several neurological disorders *via* its specific receptor Mas (Regenhardt et al., 2013; Bader et al., 2014). Besides, other bioactive components of RAS also play a part in the CNS, such as the Ang II/angiotensin receptor-2 (AT2) and Ang IV/angiotensin receptor-4 (AT4). Although the precise mechanisms of them have not been thoroughly understood, it is undeniable that the central RAS could affect how the cognitive function works.

Exogenous Ang-(1–7) has been demonstrated to enhance long-term potentiation in the hippocampus, clarifying the likely role of Mas receptor activation in preserving learning and memory (Hellner et al., 2005). Furthermore, continuous intracerebroventricular administration of Ang-(1–7) was reported to ameliorate cognitive impairment and memory dysfunction in the 5×FAD mouse, which is a transgenic mouse model of AD (Uekawa et al., 2016). Nevertheless, the precise mechanisms underlying this protective effect remain unclear; they may be associated with Mas activation and the alleviation of neuroinflammation and BBB disruption. Ang-(1–7) is usually infused continuously into the CNS instead of being administered orally because of its short half-time and peptidic nature, thereby limiting its wider use in clinical and scientific applications. Therefore, Mas receptor agonists have been used in place of Ang-(1–7) in many animal studies to activate the Ang-(1–7)/Mas axis (Sumners et al., 2013).

AVE 0991 is a non-peptide analog of Ang-(1–7) with a longer half-time than Ang-(1–7) and a high specificity for the Mas receptor (Santos and Ferreira, 2006). AVE 0991 can mimic the activity of Ang-(1–7), exerting anti-inflammatory and antioxidant effects in the heart, blood vessels, and kidneys (Carvalho et al., 2007; Silveira et al., 2013; Jiang et al., 2018). However, the effects of AVE 0991 on dNCR after surgery have not been investigated to date. Therefore, in the present study, we used AVE 0991 to activate the Ang-(1–7)/Mas axis and investigate whether AVE 0991 treatment attenuates dNCR after surgery in aged rats.

## Materials and Methods

### Chemical Reagents and Antibodies

All reagents and drugs, unless otherwise stated, were obtained from Abcam (San Diego, CA, USA). AVE 0991 was obtained from MedChem Express (Monmouth Junction, NJ, USA). The anti-cluster of differentiation 11b (CD11b) was obtained from Millipore (Billerica, MA, USA). The anti-zonula occludens-1 (ZO-1), anti-occludin, anti-claudin-5, anti-immunoglobulin G (IgG), and the secondary antibody, horseradish peroxidase-labeled goat anti-rabbit antibody were purchased from Santa Cruz Biotechnology (Santa Cruz, CA, USA). The anti-GAPDH and anti-β-actin were from Proteintech Group (Wuhan, China). The fluorescently labeled secondary antibodies were purchased from LI-COR Biosciences (Lincoln, NE, USA). Commercial enzyme-linked immunosorbent assay (ELISA) kits to determine Ang II and Ang-(1–7) levels were obtained from Cusabio Biotech (Wuhan, China).

### Animals

Aged male Sprague–Dawley rats (18 months old, 500–550 g) were used for all of the experiments. Animals were purchased from Hubei Provincial Laboratory Animal Center (Wuhan, Hubei, China) and bred under standardized housing conditions (22 ± 0.5°C, 55% ± 5% relative humidity, and a 12-h/12-h dark/light cycle) with food and water *ad libitum*. All rats were allowed at least 1 week to acclimate to the environment before the experimental manipulations. The experiment protocol was approved by the Peking University Biomedical Ethics Committee Experimental Animal Ethics Branch (approval no. LA2014-13). All efforts were made to minimize the number and suffering of the animals.

### Experiment Protocol

#### Experiment A

To study the effects of peripheral surgical trauma on RAS activity, 32 rats were randomly assigned either to a control (*n* = 8) or a surgery (*n* = 24) group and underwent laparotomy under sevoflurane anesthesia or anesthesia without surgery, respectively. Levels of the RAS components Ang II and Ang-(1–7) in the hippocampus and in blood serum were dynamically determined using ELISA at 12, 24, and 48 h after surgery (*n* = 8 per time point).

#### Experiment B

To evaluate the effects of AVE 0991 on postoperative dNCR, 66 rats were randomly assigned to control, surgery, and surgery + AVE groups (*n* = 22 per group). Rats then either received sham surgery (for the control group) or laparotomy (for surgery and surgery + AVE groups) under sevoflurane anesthesia. Rats in the surgery + AVE group received intranasal administration of 0.9 mg/kg AVE 0991 immediately after surgery. This single-point dosing protocol has been shown to enable effective penetration of a substance into the brain following intranasal administration and to protect against subarachnoid hemorrhage-induced short-term and long-term neurological deficits in rats (Mo et al., 2019). AVE 0991 was dissolved in a vehicle solution [10% Dimethyl sulfoxide (DMSO) dissolved in corn oil]. Rats in the other two groups received an identical volume of vehicle solution immediately after sham surgery or laparotomy. Spatial learning and memory were tested on the Morris water maze (MWM) task (*n* = 10 per group). Hippocampal levels of matrix metalloproteinase-9 (MMP-9) and tissue inhibitor of matrix metalloproteinase-3 (TIMP-3) were determined at 6 h after surgery using western blot and immunofluorescence (*n* = 4 per group). Besides, expression levels of Mas receptor, neuro-inflammatory molecules [interleukin-1β (IL-1β), tumor necrosis factor-α (TNF-α), CD11b, high mobility group box-1 (HMGB1), and receptor for advanced glycation end products (RAGE)], BBB tight junction (TJ) proteins (occludin, claudin-5, ZO-1) and leakage of endogenous IgG in the hippocampus were also examined at 24 h after surgery using western blot and immunohistochemistry (*n* = 4 per group). Besides, ultrastructural changes to the BBB in the hippocampal CA1 region were also assessed (*n* = 4 per group) using transmission electron microscopy (TEM).

### Anesthesia and Surgery

Brief induction of anesthesia (2% sevoflurane for 5 min) was performed in an anesthetic chamber (Li et al., 2013). Rats were then removed, placed in a supine position, endotracheally intubated, and mechanically ventilated with 2.0–2.5% sevoflurane in 100% oxygen. As described in our earlier study, the laparotomy was performed aseptically as a model of postoperative dNCR in aged rats (Li et al., 2014). Briefly, the body region undergoing surgery was shaved and sterilized. A 4-cm vertical incision was made 0.5 cm below the right costal margin. The small intestine was exteriorized approximately 10 cm and vigorously rubbed between the thumb and the index finger for 30 s. Then the small intestine was placed back in the abdominal cavity, and the incision was sutured layer by layer. Sevoflurane inhalation was stopped, the animal was extubated, and ventilation was discontinued until bodily reactions had recovered. Because the whole surgery lasted only approximately 20–25 min, arterial blood gas and blood pressure were not analyzed. The mucous color was monitored every 5 min until rats could maintain an upright posture and walk normally. Rats in the sham surgery group received identical treatment for the same duration, except that the laparotomy was not performed.

### Drug Administration

As previously described, intranasal administration of AVE 0991 was performed immediately after surgery (Mo et al., 2019). Specifically, rats were placed in a supine position under 2.0–2.5% sevoflurane anesthesia, and then 10% DMSO or 0.9 mg/kg AVE 0991 dissolved in the vehicle solution (10% DMSO dissolved in corn oil) were administered intranasally. A total volume of 36 μl was administered intranasally, by introducing 6 μl alternately into the left and right nostril every 5 min for 30 min.

### Morris Water Maze (MWM) Test

As previously described, the MWM tests were started on the first-day post-surgery (Li et al., 2013). Each rat received four training trials every day for five consecutive days. During each trial, the rats were placed in the maze facing the wall of the maze at different starting positions (northeast, northwest, southwest, and southeast). Within animals, the interval between trials was never less than 5 min. The time taken to find the hidden platform (cut-off time, 90 s) and the swimming distance were recorded. The swimming speed of each rat was calculated by the ratio of the distance and the time. On the sixth day post-surgery, the platform was removed, and each rat was allowed to swim in the maze for 90 s. The original platform site crossing times and the percentage of time and distance spent in the original platform quadrant were recorded.

### Western Blot

Western blot analyses were performed to determine the expression levels of Mas receptor, MMP-9, TIMP-3, neuro-inflammatory factors (IL-1β, TNF-α, CD11b, RAGE, and HMGB1), and BBB TJ markers (ZO-1, occludin, and claudin-5), in the hippocampus. The following primary antibodies were used: anti-Mas (1:200); anti MMP-9 (1:1,000); anti TIMP-3 (1:1,000); anti-IL-1β (1:1,000); anti TNF-α (1:1,000); anti-CD11b (1:500); anti-RAGE (1:1,000); anti-HMGB1 (1:1,000); anti-ZO-1 (1:500); anti-occludin (1:500); and anti-claudin-5 (1:500). Fluorescently labeled secondary antibodies (1:10,000) were used to detect the binding of the primary antibodies. The bound proteins were visualized by scanning the membranes in an Odyssey Infrared Imaging System (LI-COR Biosciences).

### Immunofluorescence

Tissue preparation and immunofluorescence staining of brain sections were performed as previously described (Li et al., 2013). Specifically, rats were transcardially perfused with phosphate-buffered saline (PBS; pH 7.3) followed by 50 ml 4% paraformaldehyde in PBS. The tissues were processed for paraffin embedding and 6-μm sections were prepared. The paraffin sections were deparaffinized, rehydrated, and blocked. Then the sections were incubated with antibodies against MMP-9 (1:100) and TIMP-3 (1:200). Afterward, the sections were incubated with secondary antibodies for Alexa Fluor 594 (1:500) for 1 h. The slides were imaged with a Leica Microsystems microscope (Leica, Wetzlar, Germany).

### Immunohistochemistry

As previously described (Cao et al., 2019), we used immunohistochemistry to detect extravasation of endogenous IgG in the hippocamps, which is not detectable in brain tissue under normal BBB function and used as an indicator of BBB permeability. The brain sections were incubated with the primary antibody against IgG (1:800), after which the sections were incubated with a secondary antibody, horseradish peroxidase-labeled goat anti-rabbit (1:400). The sections were imaged with a ×200 magnification using imaging software (ImagePro Plus 6.0; Media Cybernetics, Bethesda, MD, USA) for semi-quantitative analysis. The mean integrated optical density (IOD) values were analyzed.

### Enzyme-Linked Immunosorbent Assay

Levels of Ang II and Ang-(1–7) in hippocampal tissues and blood serum were determined using ELISA, as previously described (Wang et al., 2016). Blood samples were allowed to clot for 2 h at room temperature and centrifuged 1,000 *g* at 4°C for 15 min to separate the serum. Fresh hippocampal tissue was homogenized in ice-cold standard buffer containing a protease inhibitor and centrifuged at 16,000 *g* for 30 min at 4°C. The total protein concentration of the supernatant was determined using a BCA Protein Assay Kit (Beyotime, Shanghai, China). All samples were stored at −80°C until further processing. Levels of Ang II and Ang-(1–7) were measured using commercial ELISA kits, according to the manufacturer’s instructions. The sensitivities of the Ang II and Ang-(1–7) kits were 4.7–300.0 pg/ml and 18.75–1,200.00 pg/mol, respectively. Each experimental condition was tested in three wells and measured in duplicate. The optical density was measured with a microplate reader (Varioskan Flash 3001; Thermo Fisher Scientific, Waltham, MA, USA).

### Transmission Electron Microscopy

As previously described (Cao et al., 2015), hippocampal sections were washed in cacodylate buffer, immersed in osmium tetroxide solution, and stained with uranyl acetate and lead citrate. TEM (JEM-1400; JEOL, Tokyo, Japan) was used to observe ultrastructural changes in the basal laminas, TJs, and angioedema surrounding the capillaries, which are indicative of disruption to BBB integrity. Assessments were made by an independent observer blinded to the study.

### Statistical Analysis

All data are expressed as the mean ± SEM. Probe test results in the MWM studies and results obtained by ELISA and western blot were analyzed using a one-way analysis of variance (ANOVA) with multiple-comparison testing using the least significant difference (LSD) test. Escape latency and swimming speed values from the MWM were analyzed using a two-way repeated-measures ANOVA. All statistics were performed using SPSS 25.0 for MAC (IBM, Armonk, NY, USA). Statistical significance was set at *p* < 0.05.

## Results

### Laparotomy Differently Regulated the Two Axes of the Ras in the Aged Hippocampus

**Figure 1:**
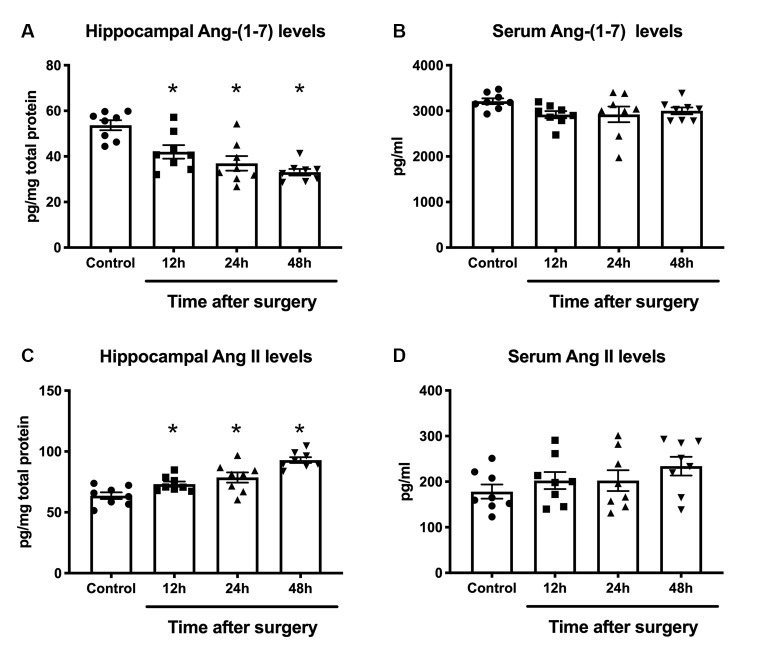
Effects of laparotomy on serum and hippocampal RAS activity in aged rats. Eighteen-month-old rats received laparotomy under sevoflurane anesthesia. Laparotomy significantly increased RAS activity in the hippocampus, leading to a decrease in Ang-(1–7) levels **(A)** and an increase in Ang II levels **(C)** but no significant changes were detected in serum RAS activity **(B,D**). Values are the mean ± SEM (*n* = 8 per group). *p < 0.05, *vs.* control group. RAS, renin-angiotensin system; Ang, angiotensin.

**Figure 2:**
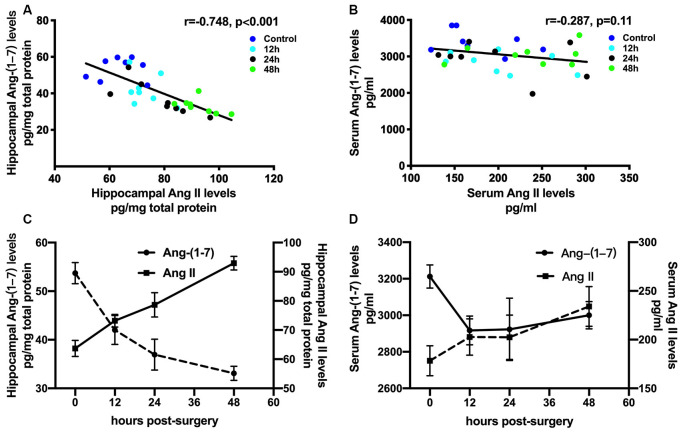
Correlations between Ang II and Ang-(1–7) levels after surgical trauma. Eighteen-month-old rats received laparotomy under sevoflurane anesthesia. Levels of Ang II and Ang-(1–7) in hippocampal tissues and blood serum were dynamically determined at 12, 24, and 48 h after surgery using ELISA. Correlation coefficients (*r*) and *P*-values were calculated using Spearman’s test. **(A,C)** In the hippocampus, the Ang II level was negatively correlated with that of Ang-(1–7). **(B,D)** Nevertheless, there was no significant correlation between levels of Ang II and Ang-(1–7) in the circulation. Values are the mean ± SEM (*n* = 8 per group). Ang, angiotensin; ELISA, enzyme-linked immunosorbent assay.

**Figure 3:**
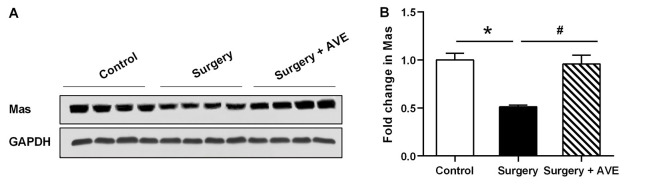
Laparotomy decreased hippocampal Mas receptor expression. Eighteen-month-old rats received laparotomy under sevoflurane anesthesia in the presence or absence of AVE 0991 treatment. Mas protein expression was determined at 24 h after surgery. Representative western blot bands **(A)** and statistical analysis **(B)** of Mas receptor expression levels. Values are the mean ± SEM (*n* = 4 per group). *p < 0.05, vs. control group; ^#^*p* < 0.05, vs. surgery group.

Ang II/AT1 and Ang-(1–7)/Mas are the important two axes of the RAS. We previously demonstrated that a surgery-induced elevation in hippocampal Ang II occurred in parallel with upregulation of AT1 protein expression (Li et al., 2014). To explore how the Ang-(1–7)/Mas axis changes after surgery, ELISA was used to detect central and circulating Ang II and Ang-(1–7) levels. As shown in Figure 1, ELISA showed that compared with the control group, Ang-(1–7) levels in the hippocampi of aged rats were significantly decreased at 12, 24, and 48 h after surgery, respectively (*p* < 0.05). Nevertheless, no significant differences in serum Ang-(1–7) levels were detected between the control and surgery groups (*p* > 0.05). The level of Ang II in the hippocampus showed an increasing trend from 12 h to 24 h to 48 h after surgery, with the values in each subgroup much higher than those in the control group (all *p* < 0.05). In contrast, serum Ang II levels were not altered at any time-point (all *p* > 0.05). There was a negative correlation between hippocampal Ang II and Ang-(1–7) levels postoperatively (Figures 2A,C; *r* = −0.748, *p* < 0.05). Nevertheless, the correlation between serum Ang II and Ang-(1–7) after surgery was not significant (Figures 2B,D; *r* = −0.287, *p* > 0.05). Furthermore, hippocampal Mas receptor expression markedly decreased at 24 h after laparotomy, while AVE 0991 treatment effectively increased Mas receptor expression (Figure 3).

### Mas Receptor Agonist AVE 0991 Attenuated Postoperative Cognitive Impairment in Aged Rats

**Figure 4:**
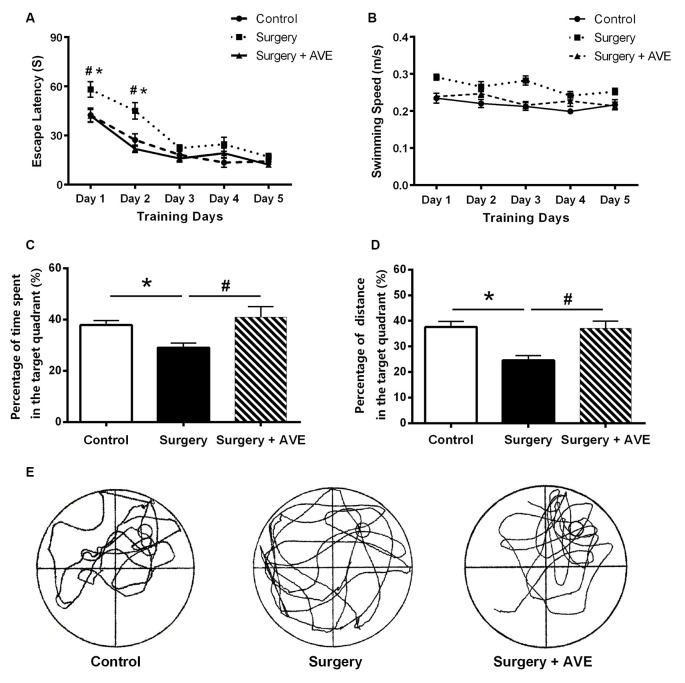
AVE 0991 treatment alleviated the hippocampal-dependent spatial memory impairment in aged rats after surgery. **(A)** Escape latency for rats to reach the platform in the navigation trials, which measures the ability of the animals to acquire spatial information. **(B)** Swimming speeds of the aged rats. **(C)** Percentage of exploration time spent within the target quadrant during probe trials. **(D)** Percentage of exploration distance within the target quadrant during probe trials. **(E)** A representative exploration trajectory. Values are the mean ± SEM (*n* = 10 per group). *p < 0.05, vs. control group; ^#^*p* > 0.05, vs. surgery group.

As shown in Figure 4, swimming speeds among groups showed no significant differences (all *p* > 0.05). The rats in the surgery group took longer to find the platform compared with those in the control group (*p* < 0.05). In the probe test, the time and distance spent in the target quadrant by rats in the surgery group were much shorter than those in the control group (both *p* < 0.05), confirming the presence of memory impairments postoperatively. All these changes were significantly attenuated by AVE 0991 treatment (all *p* < 0.05), indicating a likely cognitive protective effect of AVE 0991.

### AVE 0991 Alleviated the Hippocampal Imbalance Between MMP-9 and TIMP-3

**Figure 5:**
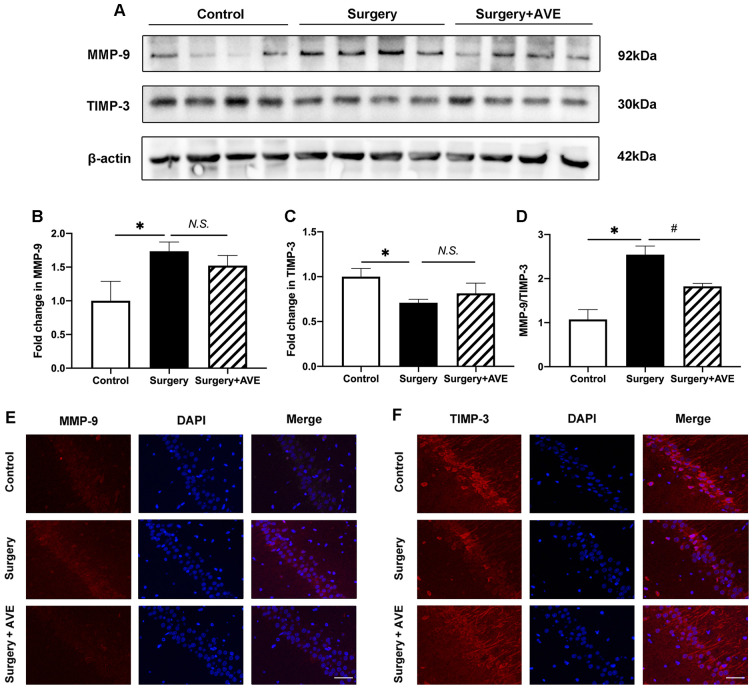
AVE 0991 treatment restored the surgery-induced imbalance in MMP-9/TIMP-3 in the aged hippocampus. Eighteen-month-old rats received laparotomy under sevoflurane anesthesia in the presence or absence of AVE 0991 treatment. Immediately after the surgical challenge, AVE 0991 was intranasally administered at a dose of 0.9 mg/kg. Representative western blot bands **(A)** and statistical analysis **(B–D)** of MMP-9, TIMP-3, and the ratio of MMP-9/TIMP-3. **(E,F)** Immunofluorescence results for MMP-9 and TIMP-3 in the hippocampal CA1 subregion at 6 h after surgery. Magnification, ×200. Scale bar, 50 μm. Values are the mean ± SEM (*n* = 4 per group). *p < 0.05, vs. control group; ^#^*p* < 0.05, vs. surgery group. *N.S*., no significance; MMP-9, matrix metalloproteinase-9; TIMP-3, tissue inhibitor of matrix metalloproteinase-3.

Our previous study found that it may be possible to correct the surgery-induced imbalance in MMPs/TIMPs by blocking the AT1 receptor of Ang II. This imbalance was most obvious in MMP-9 and TIMP-3 levels at 6 h after surgery, before the 24-h time-point (Li et al., 2016). Based on these results, we, therefore, examined hippocampal MMP-9 and TIMP-3 expression at 6 h after surgery to explore the effect of AVE 0991 on the MMP-9/TIMP-3 balance after surgery. As shown in Figure 5, MMP-9 expression significantly increased at 6 h after surgery compared with the control group (Figure 5B; *p* < 0.05), while the expression of TIMP-3 significantly decreased (Figure 5C; *p* < 0.05). Compared with the surgery group, the expression of MMP-9 in the surgery + AVE group showed a nonsignificant downward trend. Similarly, the expression of TIMP-3 increased slightly but non significantly after AVE 0991 treatment (Figures 5B,C; *p* > 0.05). Importantly, the ratio of MMP-9/TIMP-3 did show a significant decrease (Figure 5D; *p* < 0.05). We further determined the expression levels of MMP-9 and TIMP-3 in the hippocampal CA1 subregion using immunofluorescence. As shown in Figure 5E, the expression of MMP-9 in the CA1 region was clearly increased after surgery, and this effect was blocked by AVE 0991 treatment. Meanwhile, TIMP-3 expression was downregulated after surgery and showed a marked rebound in levels following AVE 0991 treatment. The lack of complete consistency between the results of the two methods may relate to differences in sensitivity and in the brain regions examined. Nevertheless, our results still suggest that AVE 0991 can effectively restore the balance between MMP-9 and TIMP-3.

### AVE 0991 Alleviated Surgery-Induced Hippocampal Neuroinflammation

**Figure 6:**
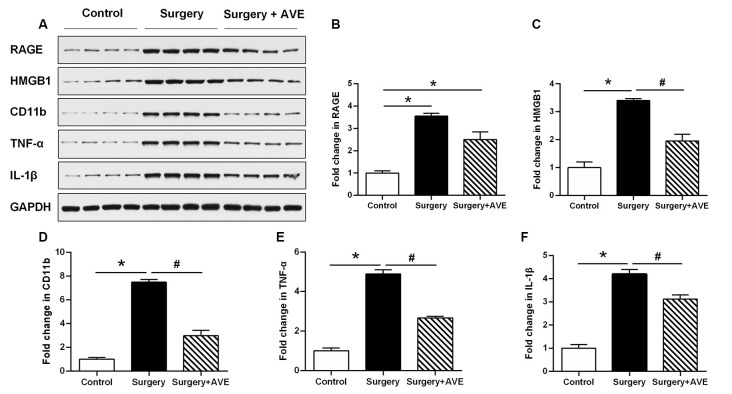
AVE 0991 treatment alleviated surgery-induced neuroinflammation in the aged hippocampus**.** Eighteen-month-old rats received laparotomy under sevoflurane anesthesia in the presence or absence of AVE 0991 treatment. Representative western blot bands **(A)** and statistical analysis **(B–F)** CD11b, an indicator of microglial activation, and inflammation-related molecules, including TNF-α, IL-1β, HMGB1, and the HMGB1 receptor RAGE. Values are the mean ± SEM (*n* = 4 per group). *p < 0.05, vs. control group; ^#^*p* < 0.05; vs. surgery group. CD11b, cluster of differentiation 11b; TNF-α, tumor necrosis factor-α; IL-1β, interleukin-1β; HMGB1, high mobility group box-1; RAGE, receptor for advanced glycation end products.

To analyze the effect of AVE 0991 on neuroinflammation in aged rats after surgery, we examined the hippocampal expression of the microglial activation marker CD11b, the inflammatory mediators TNF-α, IL-1β, and HMGB1, and the HMGB1 receptor RAGE. As shown in Figure 6, the western blot results showed that at 24 h after surgery, expression levels of CD11b, TNF-α, IL-1β, HMGB1, and RAGE in the surgery group were all significantly upregulated compared with those in the control group. Apart from the expression level of RAGE, all of these changes were significantly attenuated after treatment with AVE 0991 in the surgery + AVE group (all *p* < 0.05).

### Mas Receptor Agonist AVE 0991 Restored Hippocampal BBB Integrity in Aged Rats

**Figure 7:**
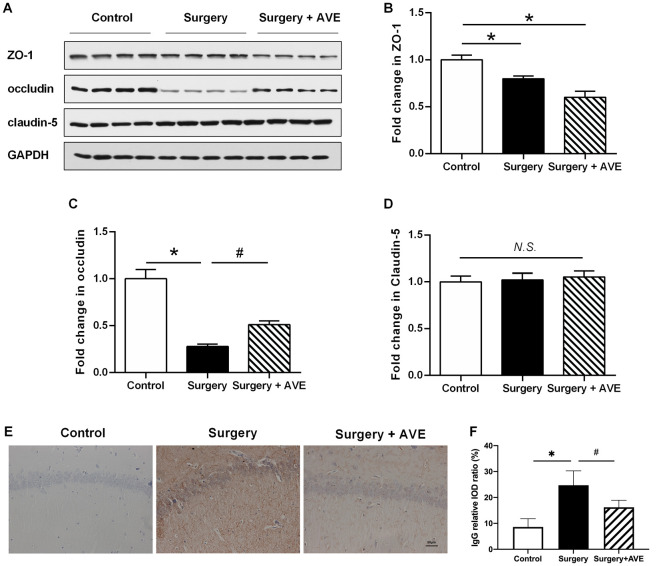
AVE 0991 treatment attenuated the surgery-induced BBB permeability. Eighteen-month-old rats received laparotomy under sevoflurane anesthesia in the presence or absence of AVE 0991 treatment.** (A–E)** The expression of the TJ proteins ZO-1, occludin, claudin-5 in the hippocampus was determined by western blot analysis. Representative western blot bands **(A)** and statistical analysis **(B–D)** of ZO-1, occludin, claudin-5. **(E)** Endogenous IgG immunohistochemical staining in the hippocampal CA1 subregion was examined (magnification × 200, scale bar, 50 μm). **(F)** The results of the semi-quantification of IgG extravasation were shown. Values are the mean ± SEM (*n* = 4 per group). *p < 0.05, *vs.* control group; ^#^*p* < 0.05, *vs.* surgery group. *N.S.*, no significance; BBB, blood-brain barrier; TJ, tight junction; ZO-1, zonula occludens-1.

To analyze the effect of AVE 0991 on hippocampal TJ protein expression levels after laparotomy, we used western blot to examine expression levels of hippocampal ZO-1, occludin, and claudin-5. As shown in Figures 7A–D, compared with the control group, expression levels of ZO-1 and occludin in the surgery group were significantly reduced at 24 h after surgery (both *p* < 0.05). Treatment with AVE 0991 significantly reversed the change in occludin expression (*p* < 0.05), while there was no significant change in claudin-5 expression in any condition (both *p* > 0.05).

To functionally analyze the effect of AVE 0991 on BBB integrity, we used immunohistochemical analysis and the IOD value ratio in the hippocampus to semi-quantificationally examine endogenous IgG extravasation. As shown in Figures 7E,F, there were marked increases in IgG leakage after surgery when compared with the control group (*p* < 0.05). Treatment with AVE 0991 significantly reduced the IgG leakage in the hippocampal CA1 region (*p* < 0.05).

**Figure 8:**
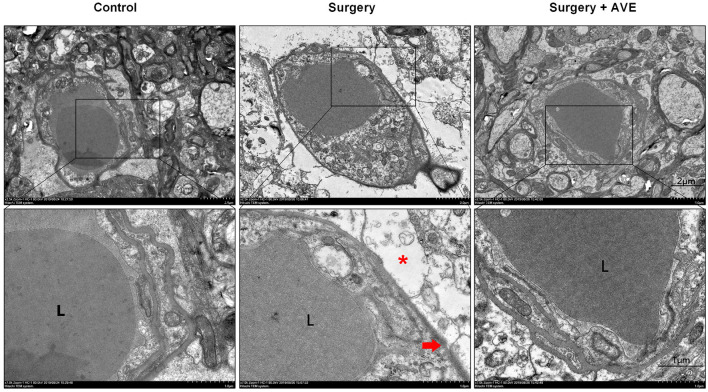
Mas activation with intranasal AVE 0991 treatment attenuated surgery-induced disruption of BBB ultrastructure**.** Eighteen-month-old rats received laparotomy in the presence or absence of AVE 0991 treatment. Disruption of BBB ultrastructure was assessed by transmission electron microscopy (TEM) of a coronal section through the hippocampal CA1 subfield (*n* = 4 per group). At 24 h postsurgery, the basal laminas had partly collapsed (red arrowhead) and the ultrastructural integrity of BBB appeared destroyed, the angioedema was evident, astrocytic end-feet (*, red asterisk) were swollen, the perivascular spaces around the basal laminas were enlarged. After AVE 0991 treatment, the pathological changes in BBB ultrastructure were alleviated, shown by a clear recovery of the basal laminas and diminished angioedema surrounding the capillaries. Scale bar, 1 μm. L, vascular cavity. BBB, the blood-brain barrier.

To further assess the effect of AVE 0991 on changes in hippocampal BBB ultrastructure after surgery, we performed TEM on hippocampal samples. In the control group, ultrastructural features of endothelial cells and TJ proteins were normal, and the basal laminas were integrated and continuous. However, the BBB basal laminas were locally collapsed and blurred, the angioedema was evident, the astrocytic end-feet around the basal lamina appeared swollen, the enlarged perivascular spaces were observed in the surgery group, suggesting that laparotomy affected the structural integrity of the BBB. AVE 0991 treatment significantly alleviated these changes in BBB integrity, as shown by a clear recovery of the basal laminas and astrocytic end-feet, as well as the diminished angioedema surrounding the capillaries (Figure 8).

## Discussion

In the present study, we assessed the neuroprotective effect of AVE 0991 in postoperative dNCR and explored the mechanisms underlying its effect. We found that laparotomy under sevoflurane anesthesia led to the activation of the hippocampal RAS, characterized by downregulation of the Ang-(1–7)/Mas axis and upregulation of Ang II. This resulted in an imbalance of MMP-9 and TIMP-3 levels in aged rats. Moreover, intranasal application of AVE 0991, a synthetic non-peptide mimic of Ang-(1–7), restored the hippocampal MMP-9/TIMP-3 balance after surgical trauma, thereby restoring BBB integrity and providing anti-inflammatory and cognition-enhancing effects. Our results are the first to establish a connection between hippocampal Ang II/Ang-(1–7) imbalance and dNCR after anesthesia and surgery.

Accumulating evidence from several animal models suggests that the central RAS participates in the modulation of cognitive function, including cognitive dysfunction induced by hypertension (Foulquier et al., 2018), diabetes (Sah et al., 2019), and stroke (Ahmed et al., 2018), as well as anesthetic surgery-induced postoperative dNCR (Li et al., 2014). The central RAS consisted of almost all components as circulating RAS with an auto-regulation mechanism (Jackson et al., 2018). The two important effector molecules of the RAS, Ang II, and Ang-(1–7), have been demonstrated to play antagonistic roles to each other in several disease processes (Iwai and Horiuchi, 2009). It is well established that Ang II *via* its AT1 receptor elicits detrimental effects while Ang-(1–7) *via* its receptor Mas exerts beneficial actions in neural pathologies (Sumners et al., 2013). A previous study from our group demonstrated that hippocampal Ang II levels increase significantly following surgery, while levels in plasma are not altered (Li et al., 2014). In the present study, we used the classical laparotomy dNCR model to explore how the level of Ang-(1–7) changes after surgery. In the current study, we found that compared with the control group, aged rats receiving laparotomy showed a significant decrease in hippocampal Ang-(1–7) levels, accompanied by a significant increase in Ang II levels within 48 h after surgery. In contrast, no significant differences in the levels of Ang II and Ang-(1–7) in serum were detected. These results show that the surgery-induced central activation of the RAS towards the detrimental direction, which was independent of changes to the circulating RAS, providing the theoretical basis of the intervention on central RAS instead of systematic treatment. Nevertheless, clinical research has revealed a decrease in plasma levels of Ang-(1–7) in AD patients compared with controls, with the decrease positively correlated with impairments in cognitive function in the patients (Jiang et al., 2016). The inconsistency between results may be because AD is a chronic disease accompanied by systemic changes, including changes to RAS activity in the peripheral circulation.

AVE 0991 has a molecular weight of 580.73 and is soluble in alkaline water solutions or organic solvents, such as DMSO or ethanol (Santos and Ferreira, 2006). In a rat model of subarachnoid hemorrhage, intranasal treatment with 0.9 mg/kg AVE 0991 dissolved in 10% DMSO was shown to attenuate oxidative stress and neuronal apoptosis (Mo et al., 2019). Take into consideration the solubility of AVE 0991, we selected the same intervention regimen (including dosage, solvent, and administration route), and similarly demonstrated that intranasal administration of AVE 0991 can ameliorate dNCR after surgery, reduce neuroinflammation, and restore BBB integrity in a Mas-dependent manner. The exact mechanisms by which drugs are delivered from the nasal cavity to the CNS are not yet thoroughly understood. However, an increasing number of studies have demonstrated that nerves connecting the nasal mucosa to the CNS (especially the olfactory nerve and the trigeminal nerve), along with the vasculature, lymphatic system, and cerebrospinal fluid circulation are important in the transport of drug molecules (Dhuria et al., 2010). In our study, hippocampal Mas expression decreased at 24 h after surgery, while AVE 0991 administration significantly increased Mas expression, though it was still lower than that in the control group. We speculate that the restoration of hippocampal Mas receptor level is an adaptive change caused by exogenous administration of AVE 0991 to activate the Mas receptor. These results demonstrate that intranasal administration of AVE 0991 can activate Mas expression in the hippocampus.

Neuroinflammation is one of the most important underlying mechanisms contributing to dNCR after surgery and manifests as an increase in inflammatory factor expression and microglial activation. Given the significant increase in the expression of the hippocampal microglial activation marker CD11b, this suggests that microglia participate in the neuroinflammatory process after surgery. Consistent with results from a splenectomy-induced model of postoperative cognitive dysfunction (Yu et al., 2014), we similarly observed that hippocampal levels of IL-1β and TNF-α increased after surgery, demonstrating that hippocampal inflammation contributes to postoperative dNCR. According to a previous study, the increase of TNF-α induced by isoflurane was mainly in neurons, while the increase of TNF-α in microglia was not so obvious (Wu et al., 2012). The result suggests neurons, like microglia, may also play a nonnegligible role in the initial and cascade stages of the inflammatory response. These inflammatory factors also switch microglia toward M1 activation, which is characterized by an enhanced inflammatory response and suppressed phagocytic activity (Franco and Fernández-Suárez, 2015). TNF-α also regulates the release of cytokines and chemokines, activates microglia, and recruits microglia to migrate toward the hippocampus (Terrando et al., 2010, 2011). HMGB1 is a widely distributed, highly conserved nucleoprotein, which takes part in intracellular DNA duplication, transcription, translation, and acts as an important extracellular late-acting cytokine. Here, we found that levels of HMGB1 and its receptor RAGE were significantly increased in the hippocampus of aged rats at 24 h after surgery, consistent with results from another rat model of postoperative cognitive dysfunction (He et al., 2012). However, intranasal administration of AVE 0991 ameliorated these changes, suggesting that AVE 0991 inhibits neuron- and microglia-induced central neuroinflammation and promotes cognitive recovery after surgical trauma.

MMPs work antagonistically with their endogenous tissue inhibitors, TIMPs, to maintain the homeostatic metabolism of extracellular matrix proteins under physiological conditions. Certain conditions induce the MMP/TIMP imbalance. Specifically, the upregulated MMPs can degrade the extracellular matrix of the basal membrane and digest TJ proteins, thus causing the BBB to leak (Vafadari et al., 2016). Our previous results demonstrated that RAS activation aggravates the imbalance between MMPs and TIMPs in the hippocampus, especially that between MMP-9 and TIMP-3, which was most imbalanced at 6 h in the laparotomy aged rat model (Li et al., 2016). Similarly, in the present study, we found that the MMP-9/TIMP-3 ratio was disrupted at 6 h after surgery, followed by remarkable IgG extravasation, decreased expression of TJ protein occludin, and BBB ultrastructure disruption at 24 h after surgery. AVE 0991 treatment could improve the MMP-9/TIMP-3 equilibrium and restore the BBB integrity in aged dNCR rats.

The degradation of the extracellular matrix is a dynamic equilibrium regulated by both MMP-9 and TIMP-3. Although the difference of expression of MMP-9 and TIMP-3 between surgery and surgery + AVE group is not significant, there is still a reversed trend by AVE 0991 and a significant difference in the MMP-9/TIMP-3 ratio. Additionally, the cognitive changes and elevation in BBB permeability were consistently observed as early as day 1 postoperatively. However, the MMP-9/TIMP-3 imbalance was most obvious at 6 h after surgery. AVE 0991 administration could restore the MMP-9/TIMP-3 imbalance at 6 h after surgery as well as the accompanied improvement in BBB structural and functional integrity at 24 h after surgery. Thus, it could be deduced that the advanced MMP-9/TIMP-3 imbalance may be an upstream mechanism of changes in BBB integrity. Collectively, it could be speculated that the surgical trauma induced RAS activation, thereby aggravating the imbalance of MMP-9/TIMP-3, which relates closely to the BBB disruption. In the current study, we employed semi-quantification of endogenous IgG extravasation into the brain parenchyma by immunohistochemistry to functionally explore the BBB permeability (Wang et al., 2012). We observed surgery-induced IgG extravasation was reversed by AVE 0991 at 24 h after surgery, supporting the conclusion that AVE 0991 treatment restored the MMP-9/TIMP-3 balance and improved BBB integrity. The leakage of plasma-derived IgG is reported to be a potent pro-inflammatory substance to activate microglia (Tucsek et al., 2014), which in turn exacerbates the surgery-induced neuroinflammation.

Nevertheless, we previously found that the AT1 antagonist candesartan effectively attenuated the surgery-induced downregulation of occludin, but did not affect the downregulation of ZO-1 (Li et al., 2016). Similarly, we herein observed that AVE 0991 attenuated the surgery-induced downregulation of occludin but not ZO-1 and claudin-5 in the hippocampus. As occludin and claudin-5 were transmembranous TJ proteins while ZO-1 was the intracellular adapter protein (Ronaldson and Davis, 2011), occludin was considered as a more likely role in TJ stability and barrier function, which was indispensable for barrier integrity in various endothelial cell models (Saitou et al., 2000). Within the endothelium, occludin was mainly proteolytically cleaved by MMPs, most MMP-2/9 and to a lesser extent MMP-3, to inactive fragments, leading to barrier disruption (Cummins, 2012). For claudin-5, it selectively decreased the permeability to ions and was regulated by several factors such as phosphorylation, cAMP, autophagy, et cetera (Jia et al., 2014; Greene et al., 2019). The different locations and functions of the TJ proteins may partly explain their different responses to surgical trauma and AVE 0991 intervention.

There are some limitations to this study. First, a control + AVE group with sham operation followed by AVE 0991 administration was not set. As for the consideration, another study had found that Mas activation by Ang-(1–7) had no significant effects on the cognitive function compared with vehicle (Janatpour et al., 2019). Thus, in terms of animal welfare, the control + AVE group was not set in the present study. Second, we did not use BBB transport tracers to functionally assess BBB permeability as we did in previous studies (Li et al., 2014, 2016), but rather only analyzed the endogenous IgG extravasation into the brain parenchyma to functionally explore the BBB permeability. Nevertheless, the surgery-induced ultrastructural changes and the improvement following AVE 0991 treatment are the same as our previous results. Therefore, we believe that AVE 0991 is effective in restoring BBB integrity.

**Figure 9:**
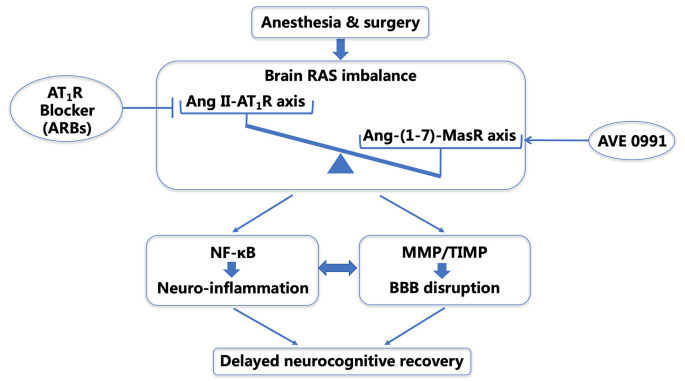
Schematic illustration of the probable mechanisms underlying delayed neurocognitive recovery (dNCR) in aged rats after anesthesia and surgery. Surgical stress activates the central RAS in the aged hippocampus, and the resultant shift in the balance of the central Ang II/Ang-(1–7) leads to NF-κB-mediated neuroinflammation and an imbalance of MMP/TIMP. This further causes BBB disruption and dNCR following surgery. Targeting the Ang II/Ang-(1–7) imbalance using ARBs (Li et al., 2016) and/or the Ang-(1–7) mimic AVE 0991 may be promising therapeutic strategies for combating postoperative neurocognitive impairment through an anti-neuroinflammatory manner and restoring the BBB integrity. dNCR, delayed neurocognitive recovery; RAS, renin-angiotensin system; Ang, angiotensin; NF-κB, nuclear factor-κB; MMP, matrix metalloproteinase; TIMP, tissue inhibitor of matrix metalloproteinase; BBB, blood-brain barrier; ARBs, angiotensin receptor blockers.

In conclusion, we found further evidence that postoperative neurocognitive impairment in aged rats is accompanied by a dysregulation of the central Ang II/AT1 and Ang-(1–7)/Mas axes. This dysregulation was associated with the inflammatory cascade and BBB disruption, which were triggered by the disturbance of the MMP-9/TIMP-3 equilibrium following a surgical challenge. Intranasal administration of AVE 0991 alleviated the MMP-9/TIMP-3 imbalance and attenuated learning and memory deficits by reducing neuroinflammation and restoring BBB integrity. The current study, as a continuation of our previous finding that ARBs targeting Ang II overactivity is beneficial for dNCR (Li et al., 2016), further suggest that targeting the Ang II/Ang-(1–7) imbalance using Ang II AT1-receptor blockers (ARB) and/or the Ang-(1–7) mimic AVE 0991 may be a promising therapeutic strategy for combating postoperative neurocognitive impairment (Figure 9).

## Data Availability Statement

The raw data supporting the conclusions of this article will be made available by the authors, without undue reservation.

## Ethics Statement

The animal study was reviewed and approved by Peking University Biomedical Ethics Committee Experimental Animal Ethics Branch.

## Author Contributions

All persons who meet authorship criteria are listed as authors, and all authors certify that they have participated sufficiently in the work to take public responsibility for the content, including participation in the concept, design, analysis, writing, or revision of the manuscript. ZL, XG, NY, and TL: conception and design of study. XM, YC, YL, YTL, JSH, YXL, and CK: acquisition of data. XM, JDH, and DH: analysis and/or interpretation of data. XM, YC, and YH: drafting the manuscript. ZL, XG, YZ, YJL, and CS: revising the manuscript critically for important intellectual content. All authors contributed to the article and approved the submitted version.

## Conflict of Interest

The authors declare that the research was conducted in the absence of any commercial or financial relationships that could be construed as a potential conflict of interest.

## References

[B1] Ahmed H. A. Ishrat T. Pillai B. Fouda A. Y. Sayed M. A. Eldahshan W.. (2018). RAS modulation prevents progressive cognitive impairment after experimental stroke: a randomized, blinded preclinical trial. J. Neuroinflammation 15:229. 10.1186/s12974-018-1262-x 30103772 PMC6090822

[B2] Bader M. Alenina N. Andrade-Navarro M. A. Santos R. A. (2014). MAS and its related G protein-coupled receptors, Mrgprs. Pharmacol. Rev. 66, 1080–1105. 10.1124/pr.113.008136 25244929

[B3] Cao Y. Li Z. Ma L. Yang N. Guo X. (2019). Isoflurane-induced postoperative neurovascular and cognitive dysfunction is associated with VEGF overexpression in aged rats. J. Mol. Neurosci. 69, 215–223. 10.1007/s12031-019-01350-8 31250275

[B4] Cao Y. Ni C. Li Z. Li L. Liu Y. Wang C.. (2015). Isoflurane anesthesia results in reversible ultrastructure and occludin tight junction protein expression changes in hippocampal blood-brain barrier in aged rats. Neurosci. Lett. 587, 51–56. 10.1016/j.neulet.2014.12.018 25524410

[B5] Carvalho M. B. Duarte F. V. Faria-Silva R. Fauler B. da Mata Machado L. T. de Paula R. D.. (2007). Evidence for mas-mediated bradykinin potentiation by the angiotensin-(1–7) nonpeptide mimic ave 0991 in normotensive rats. Hypertension 50, 762–767. 10.1161/HYPERTENSIONAHA.107.094987 17664388

[B6] Cummins P. M. (2012). Occludin: one protein, many forms. Mol. Cell Biol. 32, 242–250. 10.1128/MCB.06029-11 22083955 PMC3255790

[B7] Deiner S. Silverstein J. H. (2009). Postoperative delirium and cognitive dysfunction. Br. J. Anaesth. 103, i41–i46. 10.1093/bja/aep291 20007989 PMC2791855

[B8] Dhuria S. V. Hanson L. R. Frey 2nd W. H. (2010). Intranasal delivery to the central nervous system: mechanisms and experimental considerations. J. Pharm. Sci. 99, 1654–1673. 10.1002/jps.21924 19877171

[B9] Evered L. Scott D. A. Silbert B. (2017). Cognitive decline associated with anesthesia and surgery in the elderly: does this contribute to dementia prevalence? Curr. Opin. Psychiatry 30, 220–226. 10.1097/YCO.0000000000000321 28212172

[B10] Foulquier S. Namsolleck P. Van Hagen B. T. Milanova I. Post M. J. Blankesteijn W. M.. (2018). Hypertension-induced cognitive impairment: insights from prolonged angiotensin II infusion in mice. Hypertens. Res. 41, 817–827. 10.1038/s41440-018-0090-9 30120397

[B11] Franco R. Fernández-Suárez D. (2015). Alternatively activated microglia and macrophages in the central nervous system. Prog. Neurobiol. 131, 65–86. 10.1016/j.pneurobio.2015.05.003 26067058

[B12] Ganten D. Marquez-Julio A. Granger P. Hayduk K. Karsunky K. P. Boucher R.. (1971). Renin in dog brain. Am. J. Physiol. 221, 1733–1737. 10.1152/ajplegacy.1971.221.6.1733 4330904

[B13] Greene C. Hanley N. Campbell M. (2019). Claudin-5: gatekeeper of neurological function. Fluids Barriers CNS 16:3. 10.1186/s12987-019-0123-z 30691500 PMC6350359

[B14] He H. J. Wang Y. Le Y. Duan K. M. Yan X. B. Liao Q.. (2012). Surgery upregulates high mobility group box-1 and disrupts the blood-brain barrier causing cognitive dysfunction in aged rats. CNS Neurosci. Ther. 18, 994–1002. 10.1111/cns.12018 23078219 PMC6493557

[B15] Hellner K. Walther T. Schubert M. Albrecht D. (2005). Angiotensin-(1–7) enhances LTP in the hippocampus through the g-protein-coupled receptor mas. Mol. Cell Neurosci. 29, 427–435. 10.1016/j.mcn.2005.03.012 15950155

[B16] Iwai M. Horiuchi M. (2009). Devil and angel in the renin-angiotensin system: ACE-angiotensin II-AT1 receptor axis vs. ACE2-angiotensin-(1–7)-mas receptor axis. Hypertens. Res. 32, 533–536. 10.1038/hr.2009.74 19461648 PMC7091931

[B17] Jackson L. Eldahshan W. Fagan S. C. Ergul A. (2018). Within the brain: the renin angiotensin system. Int. J. Mol. Sci. 19:876. 10.3390/ijms19030876 29543776 PMC5877737

[B18] Janatpour Z. C. Korotcov A. Bosomtwi A. Dardzinski B. J. Symes A. J. (2019). Subcutaneous administration of angiotensin-(1–7) improves recovery after traumatic brain injury in mice. J. Neurotrauma 36, 3115–3131. 10.1089/neu.2019.6376 31037999

[B19] Jia W. Lu R. Martin T. A. Jiang W. G. (2014). The role of claudin-5 in blood-brain barrier (BBB) and brain metastases (review). Mol. Med. Rep. 9, 779–785. 3115–3131. 10.3892/mmr.2013.1875 24366267

[B20] Jiang T. Tan L. Gao Q. Lu H. Zhu X. C. Zhou J. S.. (2016). Plasma angiotensin-(1–7) is a potential biomarker for Alzheimer’s disease. Curr. Neurovasc. Res. 13, 96–99. 10.174/1567202613666160224124739 26907614

[B21] Jiang T. Xue L. J. Yang Y. Wang Q. G. Xue X. Ou Z.. (2018). AVE0991, a nonpeptide analogue of ang-attenuates aging-related neuroinflammation. Aging 10, 645–657. 10.18632/aging.101419 29667931 PMC5940107

[B22] Li Z. Cao Y. Li L. Liang Y. Tian X. Mo N.. (2014). Prophylactic angiotensin type 1 receptor antagonism confers neuroprotection in an aged rat model of postoperative cognitive dysfunction. Biochem. Biophys. Res. Commun. 449, 74–80. 10.1016/j.bbrc.2014.04.153 24814703

[B23] Li Z. Mo N. Li L. Cao Y. Wang W. Liang Y.. (2016). Surgery-induced hippocampal angiotensin II elevation causes blood-brain barrier disruption *via* mmp/timp in aged rats. Front. Cell. Neurosci. 10:105. 10.3389/fncel.2016.00105 27199659 PMC4844612

[B24] Li Z. Q. Rong X. Y. Liu Y. J. Ni C. Tian X. S. Mo N.. (2013). Activation of the canonical nuclear factor-κB pathway is involved in isoflurane-induced hippocampal interleukin-1β elevation and the resultant cognitive deficits in aged rats. Biochem. Biophys. Res. Commun. 438, 628–634. 10.1016/j.bbrc.2013.08.003 23933318

[B25] Mo J. Enkhjargal B. Travis Z. D. Zhou K. Wu P. Zhang G.. (2019). AVE 0991 attenuates oxidative stress and neuronal apoptosis *via* Mas/PKA/CREB/UCP-2 pathway after subarachnoid hemorrhage in rats. Redox Biol. 20, 75–86. 10.1016/j.redox.2018.09.022 30296700 PMC6174866

[B26] Regenhardt R. W. Desland F. Mecca A. P. Pioquinto D. J. Afzal A. Mocco J.. (2013). Anti-inflammatory effects of angiotensin-(1–7) in ischemic stroke. Neuropharmacology 71, 154–163. 10.1016/j.neuropharm.2013.03.025 23583926 PMC3664115

[B27] Ronaldson P. T. Davis T. P. (2011). Targeting blood-brain barrier changes during inflammatory pain: an opportunity for optimizing CNS drug delivery. Ther. Deliv. 2, 1015–1041. 10.4155/tde.11.67 22468221 PMC3313594

[B28] Sah S. K. Samuel V. P. Dahiya S. Singh Y. Gilhotra R. M. Gupta G.. (2019). A contemporary biological pathway of islet amyloid polypeptide for the management of diabetic dementia. Chem. Biol. Interact. 306, 117–122. 10.1016/j.cbi.2019.04.022 31004596

[B29] Saitou M. Furuse M. Sasaki H. Schulzke J. D. Fromm M. Takano H.. (2000). Complex phenotype of mice lacking occludin, a component of tight junction strands. Mol. Biol. Cell 11, 4131–4142. 10.1091/mbc.11.12.4131 11102513 PMC15062

[B30] Santos R. A. Ferreira A. J. (2006). Pharmacological effects of AVE 0991, a nonpeptide angiotensin-(1–7) receptor agonist. Cardiovasc. Drug. Rev. 24, 239–246. 10.1111/j.1527-3466.2006.00239.x 17214600

[B31] Silveira K. D. Barroso L. C. Vieira A. T. Cisalpino D. Lima C. X. Bader M.. (2013). Beneficial effects of the activation of the angiotensin-(1–7) MAS receptor in a murine model of adriamycin-induced nephropathy. PLoS One 8:e66082. 10.1371/journal.pone.0066082 23762470 PMC3676359

[B32] Sumners C. Horiuchi M. Widdop R. E. McCarthy C. Unger T. Steckelings U. M. (2013). Protective arms of the renin-angiotensin-system in neurological disease. Clin. Exp. Pharmacol. Physiol. 40, 580–588. 10.1111/1440-1681.12137 23735163

[B33] Terrando N. Eriksson L. I. Ryu J. K. Yang T. Monaco C. Feldmann M.. (2011). Resolving postoperative neuroinflammation and cognitive decline. Ann. Neurol. 70, 986–995. 10.1002/ana.22664 22190370 PMC4556354

[B34] Terrando N. Monaco C. Ma D. Foxwell B. M. Feldmann M. Maze M. (2010). Tumor necrosis factor-alpha triggers a cytokine cascade yielding postoperative cognitive decline. Proc. Natl. Acad. Sci. U S A 107, 20518–20522. 10.1073/pnas.1014557107 21041647 PMC2996666

[B35] Tucsek Z. Toth P. Sosnowska D. Gautam T. Mitschelen M. Koller A.. (2014). Obesity in aging exacerbates blood-brain barrier disruption, neuroinflammation and oxidative stress in the mouse hippocampus: effects on expression of genes involved in beta-amyloid generation and Alzheimer’s disease. J. Gerontol. A Biol. Sci. Med. Sci. 69, 1212–1226. 10.1093/gerona/glt177 24269929 PMC4172034

[B36] Uekawa K. Hasegawa Y. Senju S. Nakagata N. Ma M. Nakagawa T.. (2016). Intracerebroventricular infusion of angiotensin-(1–7) ameliorates cognitive impairment and memory dysfunction in a mouse model of Alzheimer’s disease. J. Alzheimers Dis. 53, 127–133. 10.3233/JAD-150642 27128367

[B37] Vafadari B. Salamian A. Kaczmarek L. (2016). MMP-9 in translation: from molecule to brain physiology, pathology and therapy. J. Neurochem. 139, 91–114. 10.1111/jnc.13415 26525923

[B38] Wang L. W. Tu Y. F. Huang C. C. Ho C. J. (2012). JNK signaling is the shared pathway linking neuroinflammation, blood-brain barrier disruption and oligodendroglial apoptosis in the white matter injury of the immature brain. J. Neuroinflammation 9:175. 10.1186/1742-2094-9-175 22805152 PMC3414763

[B39] Wang X. Ye Y. Gong H. Wu J. Yuan J. Wang S.. (2016). The effects of different angiotensin II type 1 receptor blockers on the regulation of the ACE-AngII-AT1 and ACE2-Ang(1–7)-Mas axes in pressure overload-induced cardiac remodeling in male mice. J. Mol. Cell Cardiol. 97, 180–190. 10.1016/j.yjmcc.2016.05.012 27210827

[B40] Wright J. W. Harding J. W. (2019). Contributions by the brain renin-angiotensin system to memory, cognition and Alzheimer’s disease. J. Alzheimers Dis. 67, 469–480. 10.3233/JAD-181035 30664507

[B41] Wu X. Lu Y. Dong Y. Zhang G. Zhang Y. Xu Z.. (2012). The inhalation anesthetic isoflurane increases levels of proinflammatory TNF-α, IL-6 and IL-1β. Neurobiol. Aging. 33, 1364–1378. 10.1016/j.neurobiolaging.2010.11.002 21190757 PMC3117127

[B42] Yu L. Sun L. Chen S. (2014). Protective effect of senegenin on splenectomy-induced postoperative cognitive dysfunction in elderly rats. Exp. Ther. Med. 7, 821–826. 10.3892/etm.2014.1501 24660030 PMC3961123

